# Linking Science to Policy: The Role of International Collaborative Research

**Published:** 2002

**Authors:** Thomas F. Babor

**Affiliations:** Thomas F. Babor, Ph.D., M.P.H., is a professor and chairman in the Department of Community Medicine and Health Care, University of Connecticut School of Medicine, Farmington, Connecticut

**Keywords:** public policy on alcohol or other drug (AOD), alcoholic beverage control system, international AOD-related (AODR) problems, history of AOD public policy, harm reduction policy, collaboration, public-private cooperative prevention, prevention research

## Abstract

This article traces the modern history of alcohol policy research on an international level, focusing on cross-national collaborative studies, a recent phenomenon that has dramatically increased our scientific understanding of how alcohol-related problems can be prevented or reduced through organized action by governments and public health organizations. The studies reviewed here show that during the past 25 years, a small but growing cadre of alcohol research professionals has used a problem-focused, integrative research approach to more closely align alcohol research with public policy.

Although governmental authorities have attempted to prevent alcohol problems since antiquity, the scientific study of such problems and the use of alcohol research to inform public policy have been rather recent developments. Not until the rise of modern medicine and the emergence of the world Temperance Movement during the 19th century was alcohol policy^1^ recognized as a potential instrument of public health, nor was epidemiological research viewed as a potential instrument of alcohol policy. During the 20th century, numerous attempts were made to employ social science techniques, such as population surveys and trend analyses of mortality data, to evaluate the effects of a wide range of policy options (Babor 1993; Edwards et al. 1994). Such policies included total prohibition, State monopolies, drinking-and-driving laws, school-based alcohol education, alcohol taxation, legislative controls on alcohol availability, age restrictions on alcohol purchasing, and media information campaigns. By the 1970s, social scientists, often under the guidance of the World Health Organization (WHO) or their national governments, began to collaborate across national boundaries to study the effects of alcohol policies on alcohol-related problems.

One major advantage of international scientific collaboration is the ability of participating scientists to compare and contrast a broad array of policy options across different countries. The comparative approach also helps in understanding the cultural idiosyncrasies of drinking and the historical determinants of alcohol policies. This article traces the modern history of international policy analysis in the alcohol field by describing a series of innovative and influential cross-national studies that have been published in the past 25 years (see table). These studies, which have been initiated at a relatively constant rate during that time period, have used a variety of approaches—ranging from clinical trials to expert opinions—to arrive at their conclusions. This review provides a global perspective on the growing sophistication of alcohol policy research and its relevance to the challenges of the 21st century.

## The “Alcohol Control Policies in Public Health Perspective” Monograph

For the purposes of this review, the modern history of international collaboration in the area of alcohol policy research began with the publication of a seminal monograph entitled *Alcohol Control Policies in Public Health Perspective* (Bruun et al. 1975)—often referred to as the “Purple Book” because of its cover. Sponsored by the WHO’s European Office, this international collaborative project brought together alcohol researchers from 13 countries who debated policy issues, evaluated original data, and critically reviewed the world literature on prevention measures. The international working group drew its scientific expertise from academic settings and research centers in the Scandinavian countries, the United Kingdom, and North America. The resulting monograph drew attention to the preventable nature of alcohol problems throughout the world and to the role of national governments and international agencies in forming rational and effective alcohol policies.

The Purple Book stimulated heated debates on alcohol problems and their prevention, not just among academics but also among policymakers and medical practitioners. The most controversial aspect of the book was its main thesis—that the average amount of alcohol consumption in a society directly affects the prevalence of problems experienced by that society. A corollary to this thesis is that one of the most effective ways to prevent alcohol problems is through policies directed at reducing average alcohol consumption, particularly by placing limits on the physical and economic availability of alcohol through such methods as restricted hours of sale and alcohol taxes. Although not inconsistent with the contemporary “harm reduction” approach, which focused attention on groups at high risk for alcohol problems (e.g., drinking drivers and chronic alcoholics) the prevention methods promoted in the Purple Book drew attention to the full spectrum of drinkers in a society, not just alcoholics. As noted by Asvall (quoted from Edwards et al. 1994): “Few books have had so much influence on the thinking and actual policy-making in this area. Its great impact was due to the authority of the group, to the very thorough way in which they did their work, and to the practical form in which they packaged their conclusions” (p. vi).

## International Study of Alcohol Control Experiences

The International Study of Alcohol Control Experiences (ISACE) project was initiated by the WHO and collaborating research institutes to continue the work first summarized in the Purple Book, which had characterized alcohol control policy as a sequential process from policy implementation to reduced drinking to fewer alcohol-related problems. According to historical observations, alcohol consumption trends between World War II and the early 1970s were surprisingly similar in Europe and North America, with the same increases occurring in countries with different economic conditions and alcohol control policies. The aim of the ISACE project was to study the dynamics of the post–World War II increase in alcohol consumption to better understand how alcohol control policies affect alcohol consumption and its adverse consequences (Mäkelä et al. 1981; Single et al. 1981; Giesbrecht et al. 1983). The project was carried out as a series of case studies in seven countries: Finland, the Netherlands, Poland, Switzerland, Ireland, Canada, and the United States. The 18 project investigators functioned as a scientifically autonomous body but benefited from the support of and collaboration with the WHO’s European Office.

Despite the diversity in drinking patterns and alcohol-related problems across the seven countries, the study supported the main thesis of the Purple Book—namely, that the post-war changes in the overall consumption of alcoholic beverages had a direct bearing on the increased prevalence of alcohol-related problems. Specifically, six conclusions emerged from the study:

- Preventive considerations carry little weight in the formation of economic policies governing the availability of alcohol.
- With increasing acceptance of drinking in the everyday life of Western societies, public policies tend toward control of excessive drinking in the individual (e.g., through treatment or punishment) rather than toward population-based approaches.
- Because alcohol availability and control involve complex cultural, social, and political phenomena, policy changes should be made with caution and with a sense of experimentation to determine their effectiveness.
- Event-based drinking problems, such as alcohol-related traffic crashes and violence, will continue to occur frequently in industrialized societies, requiring risk-reduction measures directed at both the drinking environment and the excessive drinker.
- The health and social costs associated with alcohol should be taken into account in the development of international trade agreements.
- Governments should carefully reconsider the policy of granting fiscal incentives to support the promotion and distribution of alcoholic beverages.

**Table:** International Collaborative Projects by Year of First Publication

Year of First Publication	Project	Methods
1975	Alcohol Control Policies in Public Health Perspective	Literature reviews; alcohol statistics
1981	International Study of Alcohol Control Experiences	Case studies
1984	Community Response Study	Population surveys; agency interviews
1986	State Monopolies and Alcohol Prevention	Case studies; alcohol consumption statistics
1987	International Studies in the Development of Alcohol Treatment Systems	Case studies; treatment statistics
1989	The Primary Care Project: Identification and Management of Alcohol-Related Problems	Clinical studies; health services research
1994	The Alcohol and Public Policy Project	Literature reviews; alcohol statistics
1998	Drinking Patterns and Problems in Developing Societies	Case studies; alcohol statistics; qualitative data
2000	The European Comparative Alcohol Study	Literature reviews; qualitative analyses
2000	Supply Side Initiative	Literature reviews; case studies

## The Community Response Study

A new kind of international collaborative research was initiated in 1976 under a project coordinated by the WHO and sponsored by the governments of the United Kingdom, Mexico, Zambia, and the United States (Ritson 1985; Rootman and Moser 1984). Combining social survey research with community organization techniques, the WHO Community Response Study focused on formal and informal responses to alcohol-related problems in both rural and urban communities in Mexico, Zambia, and Scotland. In the first phase of the project, both the general population and the staff and clientele of a wide variety of health and social agencies were surveyed with respect to alcohol use, alcohol-related problems, and community responses, revealing both similarities and differences among these diverse countries. For example, in all three countries, men drank more than women, and the police devoted a major part of their time to managing people with alcohol-related problems. However, rates of abstention were much higher in Mexico and Zambia than in Scotland. Drinking by adolescents was tolerated more in Scotland, whereas alcohol-related problems were more apparent in Mexico and Zambia. In Scotland, specialized services (e.g., alcoholism treatment programs and counseling) played a major role in managing alcohol-related problems, whereas few such services existed in Mexico and Zambia, and other sources of help (e.g., family members and native healers) were more likely to be mentioned in those countries.

In the second phase of this project, community intervention projects were conducted in the three first-phase sites (Room 1990). For example, in Edinburgh, Scotland, the project contributed to the planning of prevention activities by an ongoing council of community agencies. In Mexico, the project gave impetus to the formation of an Al-Anon group for wives of heavy drinkers. And at the international level, the project developed a practical manual for community planning and action for use by the WHO member States (Rootman and Moser 1984).

At best, the attempt to translate local research on alcohol-related problems into community action proved to be more difficult than anticipated, especially in relation to the development of meaningful community responses. Nevertheless, the Community Response Study showed how coordinated international research activities could be conducted within the boundaries of limited available national resources, and how this research data could be relevant to local, national, and international alcohol policies (Rootman and Moser 1984). The study generated some of the first comparative data collected through identical population surveys in different countries. It also suggested that coherent responses to alcohol-related problems require adequate epidemiological data; joint planning by a wide variety of community leaders; and community-based, culturally specific responses. Overall, the Community Response Study can be seen as the forerunner of a whole tradition of international comparative research meetings on evaluated community action projects in the alcohol field (Giesbrecht et al. 1990), suggesting that the value of this project goes far beyond its initial scope.

## State Monopolies and Alcohol Prevention

Initiated in 1986, this project focused on the potential contributions of State monopoly systems to the control of alcohol-related problems (Kortteinen 1989). The purposes of State monopolies include curbing private profit, providing tax revenues to the State, protecting domestic industries, promoting exports, safeguarding the quality of alcoholic beverages, and reducing the prevalence of alcohol-related problems. The project, which included seven developed countries and eight developing countries, was a joint venture between the WHO and two Finnish organizations, the Social Research Institute of Alcohol Studies and the Finnish Foundation for Alcohol Studies.

The investigators found that State monopolies vary considerably in form and scope, covering both the production and the distribution of alcoholic beverages, in the following ways: First, in some countries, the monopolies apply only to certain alcoholic beverages; in other countries, they apply to all such beverages. Second, regarding the effectiveness of monopoly systems, the data suggest that the more strongly a State intervenes (i.e., the more extensive a State monopoly is), the lower the per capita consumption of alcohol will be. Third, although most monopolies were established with public health objectives in mind, their success as a specific form of alcohol control differs from one country to another and presupposes public support. Control measures that do not enjoy public support may produce undesirable effects, such as the generation of a black market. Fourth, stable political conditions and the structure of a country’s political system are important. Fifth, in many countries, religion plays a role. For example, Catholicism has been implicated in the heavy drinking patterns of the wine-producing countries of Southern Europe, whereas Islam has had an influential role in restricting alcohol use in the Muslim countries (Edwards et al. 1994). And sixth, developing countries can never “copy” a developed society’s alcohol-control system. Alcohol policies have to be tailored to suit the history and culture of each particular country.

## International Studies in the Development of Alcohol Treatment Systems

In 1984, an international conference held in Sweden discussed societal responses to alcohol problems, including the development of treatment systems. The conference identified intriguing differences in the ways various countries managed alcohol problems, and led to the formation of an international collaborative group of social scientists devoted to the exploration of the comparative and structural aspects of alcoholism treatment systems. The main product of this project was a book entitled *Cure, Care, or Control: Alcoholism Treatment in Sixteen Countries* (Klingemann et al. 1992). The 32 collaborating investigators reviewed the “cure” of alcoholism in health care systems, the “care” of problem drinkers in social welfare agencies, and the “control” of problem drinkers in the criminal justice system.

The study found that after World War II, the societal view of alcoholism treatment had shifted from a “moral” concept to an “illness” concept, with many countries also mixing these concepts into a newer “alcohol problems” approach. In most countries, the general boost in treatment resources was associated with increasing deinstitutionalization (i.e., from inpatient to outpatient settings), decentralization to community-based services, and differentiation into a greater variety of services. For example, outpatient treatment became relatively more important than inpatient treatment in almost all countries. In addition, the demographic profile of service recipients shifted from an overrepresentation of the lower social strata to growing numbers of middle-class clients.

A major finding was the wide disparity in treatment services, even among developed countries. Remarkably, the wine-producing countries, which have the highest per capita alcohol consumption, were found to have smaller treatment systems (e.g., fewer resources or treatment programs). Spirits-drinking countries with relatively lower per capita consumption invested the most in alcoholism treatment services. Overall, no simple relationship existed between the density of treatment services and the amount of alcohol problems in a given country.

## The Primary Care Project

During the 1980s, several interrelated projects were initiated by the WHO to provide a scientific basis for a public health approach to alcohol screening and brief intervention in primary care settings. Recognizing the importance of primary care to the delivery of health services in much of the world and the relative absence of specialized services for treating alcohol dependence, the WHO commissioned a study (Saunders et al. 1993) to develop an international screening test that could be used in different countries. The project was inspired by the notion that once a standardized screening test was developed for use in primary care settings, it would stimulate early intervention, thereby reducing the burden of alcohol problems in different societies, including those without a specialized treatment system.

Working with alcohol researchers in Norway, Bulgaria, the United Kingdom, Mexico, Australia, and the United States, the WHO sponsored a validation study of different screening procedures that led to the development of the Alcohol Use Disorders Identification Test (AUDIT) (Saunders et al. 1993). Unlike previous screening tests, which were designed primarily to identify alcoholics, the AUDIT was designed to detect hazardous and harmful drinkers as well as people with alcohol dependence. Since its publication in 1989, the 10-question AUDIT has generated a large body of methodological and epidemiological research (Allen et al. 1997) and has become one of the most widely used alcohol screening tests in the world.

In a second phase of this project, a multicenter clinical trial of brief intervention procedures was carried out in primary health care settings in Costa Rica, Australia, the United Kingdom, Norway, Mexico, Kenya, Bulgaria, and the former Soviet Union (WHO Brief Intervention Study Group 1996). The study showed that brief interventions could significantly affect both consumption and intensity of drinking, with 5 minutes of simple advice working as effectively as 20 minutes of counseling. The findings suggested that in a population of heavy drinkers, behavior change is more a function of motivational factors and social influence than of the moderation skills and social learning techniques typically used in behavioral self-control training. The overall decline in alcohol consumption following the intervention was not attributable solely to the patients who achieved an abstinence goal or who gave up daily drinking. Rather, changes appeared to be distributed across a broad spectrum of the drinkers who reduced their consumption by small but clinically meaningful amounts.

Building on these WHO initiatives in instrument development and applied research in primary care, efforts are now being made to evaluate dissemination strategies at the community and national level in a large number of countries (Heather 1998). This phase of the project developed procedures to overcome barriers to screening and brief intervention at the level of the individual physician, allied health personnel, and the health care system itself. Such barriers include lack of time, training, and knowledge. Through the involvement of collaborating investigators from more than 20 countries over the course of two decades, the Primary Care Project has demonstrated how clinical research can directly influence the early identification of alcohol problems in organized systems of medical care.

## The Alcohol and Public Policy Project

Two decades after the publication of the Purple Book, a similar project was commissioned by the WHO to update the world literature pertaining to alcohol policy. The new study produced *Alcohol Policy and the Public Good*, a book that proved to be as controversial and thought-provoking as its predecessor (Edwards et al. 1994). Published in 1994 and subsequently translated into 8 languages, it was prepared by an independent group of 14 scientists from 9 countries who worked together in collegiate fashion over an intensive 3-year period. The project was conducted in close collaboration with the WHO’s European Office to bring public health researchers into the realm of policy analysis through literature reviews, conference presentations, original research, and consultations to national governments.

The book was derived from the preparation of background papers (Holder and Edwards 1995), group discussions, and the interim work of a small editorial team, all of which led to the authors’ consensus regarding the scientific basis of alcohol policy on an international level. The book concluded that public health policies on alcohol have come of age because of strong evidential underpinnings derived from the scientific research that has grown in breadth and sophistication since 1975. After reviewing the evidence on alcohol taxation, environmental control measures, drunk-driving countermeasures, school-based education, community action programs, and treatment interventions, the project participants arrived at two major conclusions:

- The research establishes that public health measures of proven effectiveness are available to curb the widespread costs, health consequences, and social problems related to alcohol use.
- It is appropriate to employ two intrinsically complementary approaches: a) responses that influence per capita alcohol consumption and aggregate-level problems, and b) policies targeted at specific drinking contexts and behaviors.

*Alcohol Policy and the Public Good* not only established a scientific basis for regional policymaking, such as the European Alcohol Action Plan (World Health Organization 2000) but also provided an objective analysis on which to build globally relevant policies.

In the short period since this book was published, new developments have occurred in the epidemiology of alcohol problems, particularly in the emerging states of the former Soviet Union and in countries of the developing world. With the growth of the knowledge base and the maturation of alcohol research, a new phase of the project, starting in 1998, has continued to evaluate the epidemiology of alcohol-related problems and the influence of evidence-based alcohol policies on public health (Babor 2000). In a new epidemiological analysis conducted for the second phase of the Alcohol and Public Policy Project, Rossow (2000) evaluated cross-national data on the social costs of alcohol, showing that both heavy drinking over time and acute intoxication on specific drinking occasions increase the risk of suicidal and violent behavior. In another study, Brady (2000) reviewed the literature on indigenous minorities living in four developed countries (United States, Canada, Australia, and New Zealand) where socioeconomic isolation has been associated with a distinct drinking pattern (i.e., binge drinking on typical occasions with virtually no moderate drinking). The analysis concluded that national alcohol policies have had a limited impact on the alcohol-related problems of indigenous people. As a consequence, native peoples have now begun to develop their own ways to control the local supply and distribution of alcohol. Finally, Graham (2000) evaluated the evidence on the effectiveness of alcohol policies designed to prevent problems within relatively circumscribed drinking environments, such as bars. Despite some promising findings to suggest that on-premise intervention programs, such as training in responsible beverage service, can be effective, the study concluded that program design, outcome evaluation, and theory development needed to be greatly improved before such environmental approaches to prevention could be promoted with confidence.

What these papers have in common with the Alcohol and Public Policy Project is their ability to critically evaluate scientific evidence and provide practical guidance on policies related to the prevention of alcohol problems in different societies.

## Drinking Patterns and Problems in Developing Societies

The analyses and arguments described in *Alcohol Policy and the Public Good* were based almost entirely on material from developed societies. Because the WHO has responsibility for public health on a global basis, it also commissioned a similar study drawing on material from developing societies. An independent group of 12 scholars began a systematic effort in 1998 to review the available literature, compile relevant databases, and develop case histories for selected countries (Riley and Marshall 1999). The volume resulting from this study (Room et al. in press) is called *Alcohol in a Changing World: Drinking Patterns and Problems in Developing Societies*.

The starting point for the report was the epidemiological finding that the consumption of alcoholic beverages is responsible for a substantial proportion of health and social problems in developing societies. The highest burden is in Latin America, where alcohol is estimated to account for almost 10 percent of all cases of disability and death. Although the burden is less in other developing regions, alcohol nonetheless accounts for a considerable amount of premature death and disability. Total alcohol consumption may be relatively low in developing countries, but a pattern of occasional heavy drinking can be associated with detrimental consequences, such as alcohol-related injuries from traffic crashes or violent behavior.

The findings of the report also suggest that with economic development and rising incomes, alcohol consumption and resulting problems are likely to increase, presenting developing nations with new challenges to adopt effective alcohol policies. Another factor likely to have an adverse influence is global trade agreements. With the growing emphasis on free trade and free markets, international institutions have pushed to dismantle existing control measures, such as State alcohol monopolies and taxes on alcohol.

Despite these trends, the report also showed that strategies are available to governments for limiting the harm from drinking. These strategies include the following:

- Persuading people not to drink or educating them about ways to limit harm
- Prescribing penalties for irresponsible drinking behavior
- Providing alternatives to drinking or to drink-connected activities
- Treating or otherwise helping people who experience problems with drinking
- Reducing the alcohol user’s risk of harm in other areas (e.g., by requiring motorcycle helmets)
- Regulating the availability of alcohol or the conditions of its use
- Working with social and religious movements oriented to reducing alcohol problems.

## The European Comparative Alcohol Study

The European Comparative Alcohol Study (ECAS) was a collaborative analysis of alcohol consumption and alcohol policies in 15 European countries. The main purpose of the project was to understand the dynamics of population changes in alcohol consumption and drinking patterns as well as the influence of different policy measures on these changes.

The ECAS project was funded by grants from the European Union and several of its member countries. Drawing on both comparative and longitudinal data collected between 1950 and 1995, two substantive issues emerged from this project. The first issue is the “homogenization” of alcohol consumption patterns within Europe, especially in relation to economic development, living conditions, and public policies. The second issue concerns the “natural” time scale of changes in alcohol consumption, which may offer a more realistic perspective on alcohol policy measures.

The term “homogenization” refers to the diminishing differences among nations in per capita consumption levels and drinking patterns (Leifman 2000). Between 1950 and the mid-1970s, a homogenization of beverage preferences was observed in the Mediterranean wine-drinking countries, the Central European beer-drinking countries, and the spirits-drinking countries of Northern Europe. In each group of countries, the dominating beverage lost some of its popularity. Since the mid-1970s, the process of homogenization has slowed down, although it has been sustained by a continuing reduction of wine drinking in the Mediterranean countries. Homogenization of per capita alcohol consumption has followed a similar two-step process, with the most dramatic changes taking place between 1950 and 1975. Despite homogenization, however, significant differences between the countries remain, and many qualitative features of drinking patterns seem to be persistent and immune to change, such as the pattern of weekend binge-drinking in the Scandinavian countries (Simpura and Karlsson 2000).

The ECAS has also produced a time series analysis of alcohol consumption between 1950 and 1995 in relation to various forms of mortality in 14 countries (Norström 2001). These analyses show that an additional liter of per capita alcohol consumption has a more adverse effect in the binge-drinking countries of Northern Europe than in the steadier drinking countries of Southern Europe, suggesting the importance of drinking patterns in any consideration of alcohol policies.

According to Simpura (2000), the ECAS findings show that alcohol-related phenomena generally change slowly. Changes in alcohol consumption in the European countries take place over generations, rather than over a decade or several years. Furthermore, alcohol consumption is affected not only by alcohol policies but also by a broad array of economic and social forces. This time frame makes it difficult to implement policy interventions that will have an immediate effect on drinking patterns or alcohol-related problems. Finally, the ECAS analysis of alcohol-related harm indicates that the link between per capita alcohol consumption and alcohol-related harm is not disappearing, although it is much more nuanced and complicated than suggested in earlier interpretations of this relationship.

## The Supply-Side Initiative

From a public health perspective, it is important to understand the role of alcohol supply at the local, national, and international levels and its contribution to drinking and alcohol problems. The Supply-Side Initiative (SSI) was devoted to the study of the relationship between the supply of alcohol, the amount of alcohol consumed, and their joint connection with the prevalence of alcohol-related problems. The project was particularly interested in the analysis of cross-national investments to build alcohol production facilities in developing countries in Africa and South America. The project also focused on the rapid increase in alcohol supply in the Baltic countries and Russia and on the impact of international trade agreements on the regulation of alcohol importation within countries. The SSI was conducted by a group of independent researchers from the United States, Norway, Finland, and Sweden.

During the first phase of the project, a series of articles was commissioned to provide a comprehensive review of the scientific knowledge about the alcohol supply system. The articles describe the production, distribution, marketing, and international trade of alcohol and the relationships of these factors to alcohol consumption. For example, Jernigan (2000) reviewed global commodity chains and analyzed the dynamics of power and profit making in globalized production networks. These networks, which are made up of multiple firms and occur in many national settings, account for a substantial portion of the alcohol supply in developing countries. Particularly in the brewing and distilling industries, a small number of transnational firms now control networks of local producers, importers, advertisers, and distributors. The networks have proceeded to embed transnational brands in the local culture, using product design and marketing techniques to influence local drinking practices. Thus, standard European and American bottled beers are now promoted in developing countries as the beverage of choice for discriminating drinkers rather than the traditional, low-alcohol–content beers that are brewed locally. The review concluded that globalization and rapid social changes, especially the standardization of products across national borders, have had a major influence on the alcohol supply in developing countries.

Similarly, Grieshaber-Otto and colleagues (2000) investigated the influence of international trade agreements on alcohol consumption and alcohol problems. The article concluded that a fundamental incompatibility exists between government efforts to minimize the harm associated with alcohol, particularly by regulating its supply, and international commercial treaties that promote the free flow of goods, services, and investments. The analysis also suggests that the North American Free Trade Agreement (NAFTA) and the World Trade Organization agreements present new challenges to local and national alcohol control measures. The implications of these agreements for public health have rarely been considered. The alcohol prevention measures considered the most effective (Edwards et al. 1994)—restricting the number and locations of retail outlets, taxing alcohol, and regulating beverages according to alcohol strength—are the ones most likely to be neutralized by these trade agreements.

Edwards and Holder (2000) assessed the overall relevance of supply-side issues. According to their analysis, supply-side issues are relevant to public health at every level, from the influence of drinking outlets on local communities to the impact of globalization on worldwide drinking levels. Most likely, general principles of supply side economics also drive the alcohol supply. For example, the pressure for increased profits and market share often constitutes the major influence on decisionmaking. Because of alcohol’s effects on traffic crashes, chronic illness, social and family problems, public disorder, and crime, public health researchers are beginning to look at alcohol as one of many harmful substances, rather than considering alcohol to be “just another commodity” (Edwards and Holder 2000).

## Evolving Views of Alcohol Policy

Public policies are authoritative decisions made by governments through laws, rules, and regulations (see Longest 1998). The word “authoritative” indicates that the decisions come from the legitimate purview of legislators, judges, and other public officials and not from private industry, public interest groups, nongovernmental organizations, or other entities. Generally, alcohol policies are directed at populations (e.g., underage drinkers or pregnant women) and organizations (e.g., health systems) particularly affected by alcohol as well as at individual drinkers.

From the perspective of this review, the central purpose of alcohol policy is to enhance health by reducing the harm caused by alcohol. The pursuit of health is one of modern society’s most highly cherished values, which may account for the growing interest in alcohol policy research. However, public health often competes with such economic priorities as free trade, open markets, and the promotion of alcohol as a profitable commodity. This may explain why the alcoholic beverage industry has begun to support policy studies through sponsorship of social aspect organizations, such as the Amsterdam Group and the International Center for Alcohol Policies (McCreanor et al. 2000; Babor et al. 1996). These organizations tend to favor prevention efforts that are oriented toward information and education, the least effective evidence-based policies (Giesbrecht 2000).

Until the 1970s, the scientific evidence for the prevention and treatment of alcohol problems was inadequate to serve as a basis for alcohol policymaking. The studies reviewed in this article, however, indicate that progress has been made in the past 25 years both in practical knowledge and in the process of scientific discovery. Using what some analysts have called a problem-focused integrative research approach, the projects presented here typically include a programmatic sequence of empirical studies and literature reviews organized around a common theme. Because they last between 5 and 15 years, they do not fit neatly into the traditional category of an investigator-initiated clinical study. Rather, these projects rely heavily on population-based public health sciences, such as epidemiology and health economics, with the main impetus coming from interdisciplinary, cross-national collaboration among social and behavioral scientists. A final ingredient of these projects is their independence, autonomy, and scrupulous avoidance of even the appearance of conflict of interest.

Although it is difficult to generalize from such a diverse group of studies, they have identified several important themes:

- Alcohol policies that affect drinking patterns by limiting access, discouraging driving under the influence, and reducing the legal purchasing age are likely to reduce the harm linked to specific drinking patterns.
- Health policies have a major impact on alcoholism treatment and clinical preventive services available to people within a country or region through health care financing and the organization of the health care system itself. No clear relationship exists, however, between the type and density of services and the prevalence of alcohol-related problems.
- Individual approaches to prevention (e.g., school-based prevention programs) have a much smaller effect on drinking patterns and problems than do population-based approaches that affect the drinking environment and the availability of alcohol (Edwards et al. 1994).
- Alcohol policy is rarely dictated by scientific evidence, despite major advances in the understanding of drinking patterns, alcohol-related problems, and policy interventions. Some notable exceptions to this statement include research-based policies related to minimum purchasing age and blood alcohol levels for impaired drivers.
- There seems to be a fundamental incompatibility between the economic and political values of free trade, unfettered marketing, and open access to alcohol, on the one hand, and the public health values of demand reduction, harm reduction, and primary prevention, on the other hand.

Alcohol policies are often based on a combination of political expediency, commercial interests, common sense, and public safety. Of all the factors that could influence alcohol policy, scientific research is perhaps the most important, but least influential, route to minimizing or preventing alcohol-related problems. Nevertheless, the projects summarized in this article demonstrate the value and potential of international collaborative research that is oriented toward meaningful policy issues.

## Footnotes

1 For a definition of this and other terms used in this article, see the glossary, p. 72.

## GLOSSARY

Alcohol control: Any government measure that relates to the purchase, production, or trade in alcoholic beverages, regardless of the aims of such measures.
Alcohol-related problems: Any of the range of adverse accompaniments of drinking alcohol, including medical, social, and psychological consequences. It is important to note that “related” does not necessarily imply causality.
Alcohol policy: Measures designed to control the supply of and/or affect the demand for alcoholic beverages in a population (usually national), including education and treatment programs, alcohol control, and harm-reduction strategies. Implying the need for a coordination of governmental efforts from a public health and/or public order perspective, the term originated in the Scandinavian countries and has spread widely since the 1960s.
Alcohol supply: The production, distribution, import, export, and retail sale of alcoholic beverages.
Alcohol treatment system: A set of organizations, agencies, programs, and referral channels that offers people treatment or help for alcoholism, alcohol dependence, or alcohol-related problems.
Developing societies: Countries in the Americas south of the United States, all countries in Asia except Japan and Russia, all countries in Africa, and the island states of Oceania except for New Zealand. Also included within this frame is what has sometimes been called the “fourth world”—the partially autonomous societies of indigenous peoples that are located within developed societies.
Harmful drinking: A drinking pattern that results in medical or psychological problems.
Hazardous drinking: A pattern or amount of alcohol consumption that poses risks to the drinker or others.
Per capita consumption: The average amount of pure alcohol (usually estimated in liters) consumed during a given time period (e.g., one year), calculated by dividing the total amount of pure alcohol consumed during that time by the total number of people in the population, including children and abstainers.
World Health Organization: A United Nations agency established in 1948 to protect and promote the health of member states through public health measures and relevant policy research. In addition to the WHO’s headquarters in Geneva, there are 7 regional offices, including the Pan American Health Organization, based in Washington, DC.
